# No association between major congenital malformations and exposure to Kampo medicines containing rhubarb rhizome: A Japanese database study

**DOI:** 10.3389/fphar.2023.1107494

**Published:** 2023-03-22

**Authors:** Satoko Suzuki, Taku Obara, Tomofumi Ishikawa, Aoi Noda, Fumiko Matsuzaki, Ryutaro Arita, Minoru Ohsawa, Nariyasu Mano, Akiko Kikuchi, Shin Takayama, Tadashi Ishii

**Affiliations:** ^1^ Department of Education and Support for Regional Medicine, Tohoku University Hospital, Sendai, Japan; ^2^ Department of Kampo Medicine, Tohoku University Hospital, Sendai, Japan; ^3^ Department of Pharmaceutical Sciences, Tohoku University Hospital, Sendai, Japan; ^4^ Division of Molecular Epidemiology, Department of Preventive Medicine and Epidemiology, Tohoku Medical Megabank Organization, Tohoku University, Sendai, Japan; ^5^ Laboratory of Clinical Pharmacy, Tohoku University Graduate School of Pharmaceutical Sciences, Sendai, Japan; ^6^ Department of Obstetrics and Gynecology, Tohoku University Hospital, Sendai, Japan

**Keywords:** traditional medicine, rhubarb, Kampo medicine, pregnancy, database

## Abstract

Traditional Japanese (Kampo) medicines containing rhubarb rhizome are prescribed for constipation during pregnancy; however, detailed safety information of their use for pregnant women is lacking. The aim of current study was to clarify the association between prescription Kampo-containing rhubarb rhizome (KRR) in the first trimester of pregnancy and congenital malformations in newborns. Using a large Japanese health insurance claims database, we included pregnant women who enrolled the same health insurance society from 3 months before pregnancy to the delivery date, who gave birth between 2010 and 2019, and those with data related to their infants. Pregnant women who were prescribed magnesium oxide (MgO), commonly used for constipation, during the first trimester of pregnancy and their infants were extracted as controls. Associations between KRR prescribed in the first pregnancy trimester and major congenital malformations (MCM) in the infants were examined using multivariate logistic regression analysis. Of 75,398 infants, 4,607 (6.1%) were diagnosed with MCMs within the first year after birth. Furthermore, 9,852 infants were born to women prescribed MgO, among whom 680 (6.9%) had MCMs; 450 infants were born to women prescribed KRR, among whom 28 (6.2%) had MCMs. Multivariate logistic regression analysis identified no difference in MCM risk between the two types of prescriptions [crude odds ratio (OR) 0.895, 95% confidence interval (CI) 0.606–1.322, adjusted OR 0.889, 95% CI 0.599–1.320]. In conclusion, the risk of MCMs did not differ between those prescribed KRR or MgO in the first trimester of pregnancy.

## 1 Introduction

Approximately 40% of pregnant women experience constipation, generally during the second and third trimesters of pregnancy (Shin et al., 2015; Samavati et al., 2017). Secretion of progesterone prolongs the transit time of food through the intestinal tract and reduces water, leading to hardening of the feces (Samavati et al., 2017). When changing lifestyle and diet does not improve symptoms, the recommended first-line therapy comprises osmotic laxatives (Ford, 2008), such as magnesium compounds. In Japan, magnesium oxide (MgO) is used as an osmotic laxative (Morishita et al., 2021). The second-line therapy is based on stimulant laxatives, mainly senna (Ford, 2008; Ács et al., 2010). Stimulant laxatives include sennosides and senna glycosides (SG). In herbal medicine, rhubarb is used as a treatment for constipation during pregnancy (Samavati et al., 2017). Generally, rhubarb rhizome is used in Japan and present in some traditional Japanese (Kampo) medicines for constipation (Iizuka and Hamamoto, 2015; Ogawa et al., 2021). Sometimes daikenchuto, a Kampo medicine without rhubarb rhizome, is also used for constipation (Tsuda et al., 2016; Arita et al., 2019; Sugimine et al., 2021; Namiki et al., 2022).

The 18th edition of the Japanese Pharmacopeia classifies rhubarb as the rhizome of *Rheum palmatum* Linné, etc., which contains not less than 0.25% of sennoside A. Rhubarb rhizome has a variety of functions; it has a laxative effect (Yang et al., 2022), improves blood flow (Lin et al., 2018), and has an anti-inflammatory effect (Hu et al., 2014). Kampo medicine formulation combines crude drugs containing various ingredients, each with its own pharmacological action. For example, rhubarb rhizome is used in Kampo medicine formulations, such as mashiningan, daiokanzoto, and otsujito.

The current drug information indicates that Kampo-containing rhubarb rhizome (KRR) are not recommended for pregnant women or women who may be pregnant (Interactions, 2013). However, the deprecation is partly based on its description in a Chinese classic textbook which refers to it as Honzokomoku, a crude drug. In experimental findings, emodin, contained in rhubarb rhizome, may induce embryonic cytotoxicity in mouse blastocysts (Chang et al., 2012). Medicines-containing rhubarb rhizome are not recommended for pregnant women according to the European Medicines Agency (EMA). Despite these reports, the evidence of their effect on pregnant women has not been well established.

Previously, we investigated the frequency of Kampo medicine prescriptions for pregnant women from extensive Japanese health insurance claims databases (Suzuki et al., 2021). We found that KRR was prescribed to approximately 1.2% of pregnant women. However, we could not determine the association between the prescription of KRR to pregnant women and adverse events in the newborn.

To our knowledge, information on the safety of KRR during pregnancy is lacking. Therefore, we used an extensive health insurance claims database to investigate associations between KRR prescribed in the first trimester and major congenital malformations (MCM) in newborns.

## 2 Methods

### 2.1 Database

We collected data from the Japan Medical Data Center Co. Ltd. (Tokyo, Japan) (Kimura et al., 2010). This stores anonymous data for privacy protection, but information including sex, the birth year and month was available. The claims data are recorded information; diagnoses defined by the International Classification of Diseases, 10th Revision (ICD-10) codes, drug prescriptions covered by health insurance at hospitals including outpatient and inpatient care, dispensing at pharmacies, and procedures including operation.

Institutional review board in Tohoku University School of Medicine approved this research on 19 July 2016 (registration number: 2016-1-230). The informed consent need was disregarded, because this dataset was de-identified.

### 2.2 Study population

We used the dataset available on 8 May 2020, for this study, which included 7,447,761 men and women covered by health insurance between January 2005 and November 2019. Insured individuals had anonymous family and personal identification numbers. Therefore, we identified mother-child relationships among individuals if their newborns were entered with the identical health insurer. In addition, this dataset allowed us to identify the birth year and month (Information about the date of birth was not contained).

**FIGURE 1:**
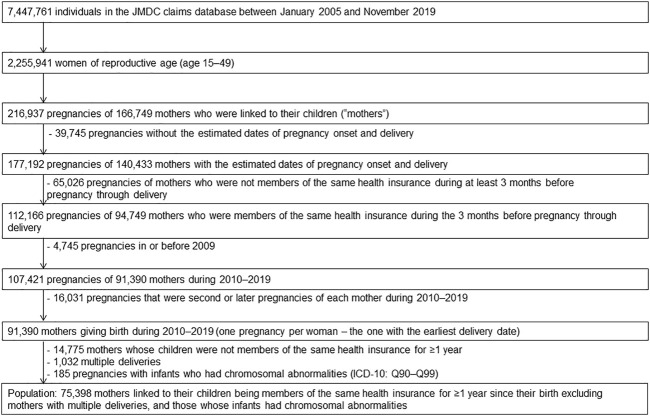
Flow chart outlining the study population selection.

The study population were defined mother who matched the following eligibility criteria; mother who linked to her infant whose birth month accorded with month of enrollment in the health insurance, mother who continued to be covered in the same health insurance from 3 months prior to pregnancy period until delivery, and mother whose dates of pregnancy onsets and delivery were estimable. Figure 1 presents a flowchart of the patient selection process. The database contained a limited number of women who gave birth before 2009; thus, we included only women who became pregnant for the first time between 2010 and 2019. Furthermore, to investigate the teratogenic risk of exposure to KRR in first trimester pregnancy, mothers whose newborns continued to be covered with the same health insurance during their birth month for more than a year since birth were included. Conversely, mothers with multiple deliveries were excluded since the risk of congenital disabilities increases with multiple births (Zhang et al., 2011). Mothers whose infants had chromosomal abnormalities (the ICD-10 codes Q90–Q99) (Huybrechts et al., 2019) were also excluded because it is not associated with exposure and their inclusion would reduce the detection of exposure-related risk.

### 2.3 Pregnancy onset and delivery date estimations

Since neither data on pregnancy onset nor delivery date were obtained in this database, we used the estimation methods reported previously (Ishikawa et al., 2018a; Ishikawa et al., 2018b; Ishikawa et al., 2019; 2020; 2022; Suzuki et al., 2021).

**FIGURE 2:**
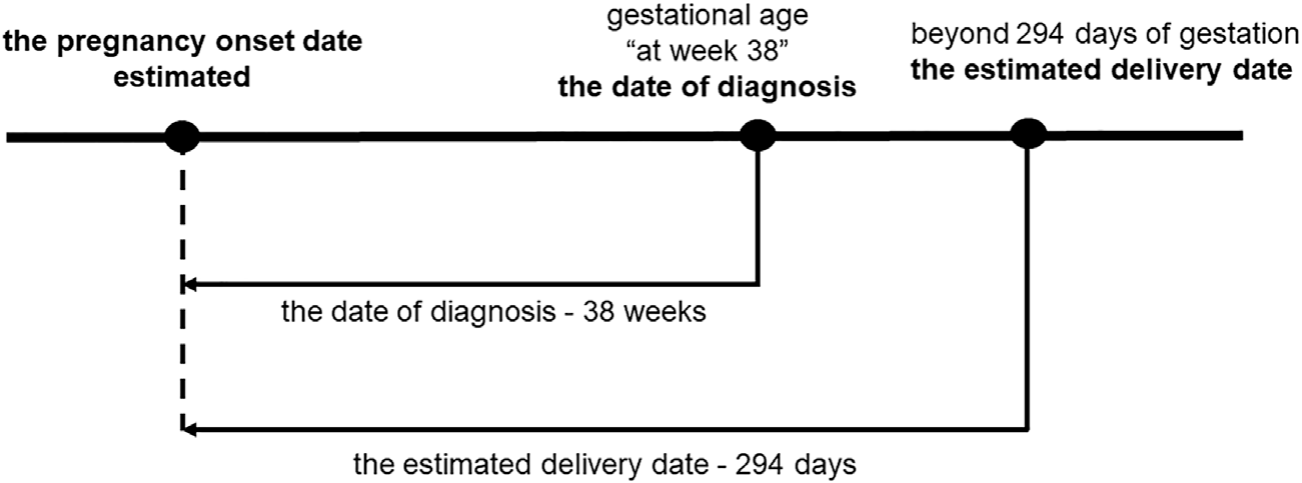
Pregnancy onset date estimations. The pregnancy onset date was estimated by subtracting the GA accompanying the diagnosis of the specific visit from the date of diagnosis. In cases for which GA was diagnosed at 38 weeks, 38 weeks and 0 days were subtracted from the date of diagnosis. When the difference between the estimated dates of pregnancy onset and delivery exceeded 294 days, pregnancy onset was considered 294 days before the estimated delivery date.

Figure 2 shows that the pregnancy onset date was calculated by subtracting the gestational age (GA) associated with the diagnosis from the date the disease was diagnosed (Ishikawa et al., 2018a; Ishikawa et al., 2018b; Ishikawa et al., 2019; 2020; 2022; Suzuki et al., 2021). In the case that a mother had several visits with diagnoses, the most recent GA was used, because a more accurate diagnosis could be made in the later stages of pregnancy (American College of Obstetricians and Gynecologists, 2017). A study based on university hospital records demonstrated that that approximately 90% of the pregnancy onset dates estimated by this method fell within ±1 week of the true pregnancy onset, indicating a high accuracy rate (Ishikawa et al., 2018a).

The delivery date was estimated by delivery-related entries and the infants birth months, using previously reported algorithm (Ishikawa et al., 2019; Suzuki et al., 2021). A study based on university hospital records demonstrated that approximately 95% of the estimated delivery dates were within ±1 week of the actual delivery date (Ishikawa et al., 2018b). In Japan, health insurance does not always cover deliveries that do not require procedures or medications. Therefore, in cases where delivery data were unavailable, the 15th day of the month of the newborn’s birth was set as the delivery date.

Induction of labor is recommended in pregnancy over 294 days, because of increased perinatal mortality (Minakami et al., 2014). When the difference between the estimated dates of delivery and pregnancy onset was more than 294 days, a gestational period was set at 294 days, and pregnancy onset was subtracted 294 days from the estimated delivery date (Figure 2). The first trimester was defined as pregnancy onset to week 13 days 6 of gestation, the second trimester as week 14 days 0 to week 27 days 6, and the third trimester as week 28 days 0 to delivery (Minakami et al., 2014).

### 2.4 Laxative prescriptions

Supplementary Table S1 lists the laxatives evaluated in this study. Pregnant women prescribed MgO during the first trimester of pregnancy and their infants were used as controls (the World Health Organization Anatomical Therapeutic Chemical Classification System (WHO-ATC) code A02AA02). Based on our previous report (Suzuki et al., 2021), we evaluated 17 KRRs prescribed to pregnant women (Supplementary Table S2). SGs (WHO-ATC code A06AB06), including sennosides and senna, were excluded from the analyses because they have the same components as rhubarb rhizome. Daikenchuto, Kampo medicine without rhubarb rhizome, was also extracted to compare with the frequency of prescriptions in KRR. The timing of exposure to the drug was adopted in preference to the day of dispensing. If the dispensing date was not available from the database, the date of admission was used. If neither the dispensing date nor the admission date could be obtained, the 15th was used as the dispensing date, because the month and year were listed on each claim. The exposure period was calculated from the days of supply for each prescription. The study extracted both regular prescriptions and laxative prescriptions as needed. The laxatives were considered as required in this study.

### 2.5 Outcomes

Supplementary Table S3 shows the selected MCMs and their ICD-10 codes. Congenital minor malformations (ICD-10 codes Q00–Q89) were excluded, and diagnoses of MCMs given to newborns during the first year of life were extracted (Ishikawa et al., 2021). In addition, the MCMs were listed separately individual organ system. A study based on university hospital records demonstrated that the MCMs in the claims were validated against medical records, and the overall positive predictive value of the MCMs was about 90% (Ishikawa et al., 2021).

### 2.6 Covariates

We considered the covariates during the adjustment for confounders; maternal age at birth, birth year, premature birth, medical history of epilepsy, diabetes, obesity, and the use of teratogenic drugs in the first trimester of pregnancy. These covariates were defined on the basis of previous papers (Eriksson et al., 2000; Ive et al., 2013; Minakami et al., 2014; Alvarado-Terrones et al., 2018; Trefz et al., 2018; Mbizvo et al., 2020). These covariates were considered a cause of exposure, of outcome, or of both or a proxy for an unmeasured factor.

### 2.7 Statistical analyses

The prevalence of laxative prescriptions during pregnancy was described and calculated for each trimester. To investigate the teratogenic risk exposure to KRR in the first-trimester, two groups were selected from the study population: pregnant women who were prescribed MgO in the first trimester of pregnancy (Group 1) and those who were prescribed KRR in the first trimester (Group 2). Women prescribed MgO and KRR were placed in Group 2. In both groups, pregnant women prescribed SG in the first trimester of pregnancy were excluded because both SG and rhubarb rhizome contain sennoside A.

Comparisons between two groups were performed using Student’s t-tests and chi-square tests for continuous and categorical variables, respectively. We performed the Cochran–Armitage test to determine trends in the frequency of laxative prescriptions in the first, second, and third trimesters of pregnancy. *p* < 0.05 indicated a significant difference. Odds ratios (OR) and 95% confidence intervals (CI) for MCM were evaluated by multivariate logistic regression analysis. We assessed the results after adjusting for multiple confounding factors. Significant differences were considered when the 95% CIs did not cross 1.0. Statistical analyses were performed using SAS version 9.4 (SAS Institute, Inc., Cary, NC, United States).

Sensitivity analyses were also performed. The analyses were repeated 1) including only women who did not receive a prescription for a suspected teratogenic drug in the first trimester of pregnancy, 2) including only pregnant women who had been prescribed laxatives for more than 30 days during the first trimester, 3) after excluding pregnant women with combined prescriptions of MgO and KRR, and, 4) after excluding pregnant women who received only a prescription as needed.

## 3 Results

### 3.1 Population characteristics

The population of this study consisted 75,398 women selected as shown in the flowchart in Figure 1. The delivery dates of 37,213 women (49.4%) were estimated using date of specific diagnoses and procedures for the aforementioned algorithms; the dates of 7,117 women (9.4%) were estimated using dates of other delivery-related entry, and the dates of 31,068 women (41.2%) were based on the 15th day of the neonatal birth month. 1,134 women (1.5%) were evenly assigned 294 days as duration of gestation, because their duration of gestation exceeded 294 days. The mean of maternal age at delivery was 32.3 years [standard deviation (SD) 4.6], and the mean of gestation period at delivery was 270.7 days (SD 12.9, lowest value 119 days, highest value 294 days). Table 1 shows the basic characteristics of the MgO-prescribed and KRR-prescribed groups during first trimester extracted from the study population.

**TABLE 1:** Study population characteristics during the first trimester of pregnancy. Statistically significant as p* <0.05 and statistically highly significant as p** <0.001.

Variables	Women with MgO prescriptions in the first trimester (*n* = 9,852)		Women with KRR prescriptions in the first trimester (*n* = 450)		*p*
	N	%	N	%	
**Maternal age at delivery**					0.136
**−24** **years**	366	3.7	20	4.4	
**25–29** **years**	2,155	21.9	85	18.9	
**30–34** **years**	3,860	39.2	174	38.7	
**35** **years**	3,471	35.2	171	38	
**Delivery year**					<.0001**
**2010–2012**	1,110	11.3	78	17.3	
**2013–2015**	3,523	35.8	181	40.2	
**2016–2019**	5,219	53	191	42.4	
**Newborn sex**					
**Male**	5,039	51.1	235	52.2	
**Female**	4,813	48.9	215	47.8	
**Hypertension**	171	1.7	21	4.7	<.0001**
**Diabetes**	447	4.5	24	5.3	0.4291
**Obesity**	66	0.7	32	7.1	<.0001**
**Epilepsy**	91	0.9	4	0.9	0.9398
**Phenylketonuria**	0	0	0	0	
**Prescription of suspected teratogenic medication in the first trimester**	37	0.4	1	0.2	0.5998
**Preterm birth**	1,165	11.8	72	16	0.0077*
**Caesarean section**	2,232	22.7	110	24.4	0.3758

Abbreviations: MgO = magnesium oxide; KRR, Kampo-containing rhubarb rhizome.

### 3.2 Prescribed laxatives from 2010 to 2019

Overall, 29,837 (39.57%) women were prescribed laxatives during their pregnancy (Table 2); MgO (*n* = 26,091, 34.60%) was the most frequently prescribed oral laxative, followed by SG (*n* = 5,542, 7.35%), KRR (*n* = 1,004, 1.33%), and daikenchuto (*n* = 387, 0.51%). In the KRR, mashiningan was prescribed most frequently (*n* = 321, 0.43%), followed by daiokanzoto (*n* = 198, 0.26%) and otsujito (*n* = 160, 0.21%).

**TABLE 2:** Prescription frequencies of laxatives during pregnancy. Trends in the frequency of prescriptions for each laxative in the first, second, and third trimesters of pregnancy. p*<0.05 indicated a significant difference. p**<0.001 indicated a highly significant difference.

Medicinal product	Total		Triemster						*p*
	(*n* = 75,398)		First (*n* = 75,398)		Second (*n* = 75,398)		Third (*n* = 75,224)		
	N	%	N	%	N	%	N	%	
All laxatives	29,837	39.57	11,958	15.86	17,838	23.66	19,843	26.38	<.0001**
MgO	26,091	34.60	10,483	13.90	16,043	21.28	16,887	22.45	<.0001**
SG	5,542	7.35	1,590	2.11	2,153	2.86	3,655	4.86	<.0001**
Daikenchuto	387	0.51	135	0.18	151	0.20	192	0.26	0.0013*
KRR									
Overall	1,004	1.33	474	0.63	460	0.61	451	0.60	0.4689
Mashiningan	321	0.43	131	0.17	174	0.23	152	0.20	
Daiokanzoto	198	0.26	97	0.13	99	0.13	95	0.13	
Otsujito	160	0.21	40	0.05	66	0.09	97	0.13	
Bofutsushosan	111	0.15	76	0.10	34	0.05	32	0.04	
Tokakujokito	74	0.10	54	0.07	14	0.02	17	0.02	
Keishikashakuyakudaioto	64	0.08	27	0.04	38	0.05	27	0.04	
Junchoto	51	0.07	23	0.03	22	0.03	23	0.03	
Crude daio	30	0.04	24	0.03	8	0.01	5	0.01	
Tsudosan	10	0.01	4	0.01	2	0.00	5	0.01	
Choijokito	8	0.01	4	0.01	2	0.00	3	0.00	
Jidabokuippo	8	0.01	3	0.00	2	0.00	5	0.01	
Daisaikoto	8	0.01	5	0.01	2	0.00	2	0.00	
Daiobotampito	5	0.01	5	0.01	0	0.00	0	0.00	
San’oshashinto	4	0.01	3	0.00	2	0.00	1	0.00	
Inchinkoto	3	0.00	2	0.00	1	0.00	0	0.00	
Jizusoippo	1	0.00	1	0.00	1	0.00	1	0.00	
Daijokito	1	0.00	1	0.00	0	0.00	0	0.00	

Abbreviations: MgO = magnesium oxide; SG, senna glycosides; KRR, Kampo-containing rhubarb rhizome.

In the first trimester of pregnancy, 11,958 (15.86%) women were prescribed laxatives. MgO (*n* = 10,483, 13.90%) was prescribed most frequently, followed by SG (*n* = 1,590, 2.11%), KRR (*n* = 474, 0.63%), and daikenchuto (a Kampo medicine without rhubarb (*n* = 135, 0.18%). Of the KRR, mashiningan was prescribed the most (*n* = 131, 0.17%), followed by daiokanzoto (*n* = 97, 0.13%) and bofutsushosan (*n* = 76, 0.1%) (Table 2).

Trends in the frequency of prescriptions in the first, second, and third trimester demonstrated significant differences between all laxatives, MgO, SG, and daikenchuto (Table 2).

### 3.3 Teratogenic risks of first-trimester exposure to KRR

Overall, 4,607 of 75,398 infants (6.1%) were diagnosed with MCMs during the first year after birth. In total, 680 of 9,852 infants born to women who prescribed MgO laxatives in the first trimester diagnosed MCMs (6.9%) and 28 of 450 infants born to women who prescribed KRR in the first trimester diagnosed MCMs (6.2%) (Table 3).

**TABLE 3:** Prevalence of MCMs in the two groups.

MCMs	Women with MgO prescriptions in the first trimester (*n* = 9,852)		Women with KRR prescriptions in the first trimester (*n* = 450)	
	N	%	N	%
MCMs overall	680	6.9	28	6.2
MCMs of the nervous system	43	0.4	2	0.4
MCMs of the eyes, ears, face, and neck	13	0.1	0	0
MCMs of the circulatory system	248	2.5	13	2.9
MCMs of the respiratory system	34	0.3	2	0.4
Cleft lip and cleft palate	15	0.2	0	0
MCMs of the digestive system	47	0.5	3	0.7
MCMs of the genital organs	44	0.4	0	0
MCMs of the urinary system	48	0.5	3	0.7
MCMs and deformations of the musculoskeletal system	216	2.2	6	1.3
Other MCMs	27	0.3	2	0.4

Abbreviations: MgO = magnesium oxide; MCM, major congenital malformation; KRR, Kampo-containing rhubarb rhizome.

KRR prescribed in the first trimester of pregnancy was not associated with a risk of MCMs in infants compared with those prescribed MgO in the first trimester (crude OR 0.895, 95% CI 0.606–1.322, adjusted OR 0.889, 95% CI 0.599–1.320). The sensitivity analyses produced similar results to those of the primary analysis (Table 4).

**TABLE 4:** Associations between the laxatives prescribed during the first trimester of pregnancy and MCM risk.

Variables	Sample size	No. of MCMs	Crude OR (95% CI)	Adjusted OR (95% CI)
Analysis adjusted for multiple confounding factors	10,302	708	0.895 (0.606–1.322)	0.889 (0.599–1.320)
Sensitivity Analysis 1: Only women who did not receive a suspected teratogenic drug prescription	10,264	707	0.895 (0.606–1.322)	0.891 (0.601–1.323)
Sensitivity Analysis 2: Only women who were prescribed laxatives for more than 30 days during the first trimester	1,495	107	1.158 (0.604–2.219)	0.968 (0.479–1.955)
Sensitivity Analysis 3: Excluded women who were prescribed both MgO and KRR	10,200	698	0.736 (0.455–1.19)	0.755 (0.466–1.225)
Sensitivity analysis 4: Excluded women who were only prescribed MgO or KRR as needed	10,185	698	0.911 (0.617–1.347)	0.905 (0.610–1.344)

Abbreviations: MgO = magnesium oxide; MCM, major congenital malformation; OR, odds ratio; CI, confidence interval; KRR, Kampo-containing rhubarb rhizome.

## 4 Discussion

To our knowledge, this novel report demonstrated the association between KRR prescriptions to pregnant women and MCMs in newborns using a large health insurance claims database. We identified no association between KRR prescriptions in the first trimester and MCMs in newborns. Our findings regarding the teratogenicity of KRR, prescribed for constipation during pregnancy, are important for counseling pregnant women prescribed KRR.

The information between KRR use during pregnancy and adverse events in infants could not be established. The components of rhubarb rhizome are sennoside A-F, emodin, aloe-emodin, and rhein (Komatsu et al., 2006). Relatedly, a population-based case-control study also found no association between senna, including sennoside A and B (included in rhubarb rhizome), and MCMs, concluding it had no teratogenic potential (Ács et al., 2010). Conversely, emodin has been reported to induce embryotoxicity in mouse blastocysts (Chang et al., 2012). Rhubarb rhizome contains diverse constituents and a considerable quantity of tannin, and it is a much more potent laxative than senna (Cirillo and Capasso, 2015). Our finding suggests that KRR, although multicomponent and having various effects, is not associated with MCMs, which was confirmed through several sensitivity analyses.

It is currently ethically difficult to investigate the safety of medicines for pregnant women, as studies investigating the safety of medicines generally exclude pregnant women. Medical administrative databases are useful for examining the safety of perinatal medicines, and are used worldwide for perinatal pharmacoepidemiological assessments. In Japan, large databases have infrequently been used to evaluate drug safety in pregnant women because information on GA is unavailable. Methods for estimating GA in Japanese administrative data have been established, allowing the identification of early pregnancy prescriptions (Ishikawa et al., 2018a). Furthermore, it was shown that reliable teratogenic risk assessment is possible in Japanese administrative data (Ishikawa et al., 2021). With the establishment of this method, the present study showed, based on a large database, that the association between KRR and the risk of MCMs in women prescribed MgO was comparable and that KRR could be a safe treatment option as a laxative during pregnancy.

In Japan, few studies on laxative prescriptions for pregnant women in large databases have been reported. In this study, laxatives were prescribed to 40% of women during pregnancy. A large database study in France reported that 15.3% of pregnant women were prescribed laxatives (Meyer et al., 2021). In this study, MgO (an osmotic laxative) was prescribed most commonly (35%), followed by SG (a stimulant laxative, 7%). It should be considered that MgO is not classified as a laxative in France, whereas laxatives are prescribed more frequently in Japan than in France.

Constipation is more common in later stages of pregnancy (Samavati et al., 2017), and all laxatives, MgO, SG, and daikenchuto were prescribed more frequently during later stages than in the first trimester. Trends in the frequency of these laxative prescriptions in the first, second, and third trimester demonstrated significant differences. Conversely, the frequency of KRR prescriptions was almost equal throughout the gestational period. Trends in the frequency of KRR prescriptions did not differ among each trimester. KRR may have been avoided because of the accompanying documentation stating that these drugs can cause miscarriages and preterm birth. In practice, infection and insufficient blood flow are the primary causes of preterm births (Suresh et al., 2022), and it remains unclear whether they can be medication-induced. In contrast, rhubarb rhizome has anti-inflammatory properties (Hu et al., 2014) that may help control infection, and its ability to improve blood flow (Lin et al., 2018) that may help reverse blood flow failure. We intend that future studies should investigate rhubarb rhizome’s broader effects using different populations.

We should acknowledge that there are limitations in this study. First, we could not confirm whether women really took the medicines that prescribed, which may result in an underestimation of risk. However, use of prescription data has the advantage of preventing recall bias related with data obtained from self-assessment. Second, laxatives are available at pharmacies as over-the-counter drugs, but data on those purchased outside the health insurance could not be obtained. Third, the study population included only mothers with data related to their infants entered in the same health insurance. Thus, evaluating the laxatives prescribed to women whose pregnancies ended in stillbirth or abortion was not possible. Finally, mother’s drinking, smoking, and other lifestyle habits may also be confounding factors; however, we could not evaluate these.

In conclusion, the risk of MCMs did not differ between women prescribed KRR and those prescribed the standard MgO laxative in the first trimester of pregnancy.

## Data Availability

The original contributions presented in the study are included in the article/Supplementary Material, further inquiries can be directed to the corresponding author.

## Abbreviations

ICD-10; International Classification of Diseases 10th Revision, MCM; Major congenital malformation, MgO; Magnesium oxide, SG; Senna glycosides, OR; Odds ratio, CI; Confidence interval, KRR; Kampo-containing rhubarb rhizome, GA; gestational age.
