# Supplementary material for: No association between major congenital malformations and exposure to Kampo medicines containing rhubarb rhizome: A Japanese database study
Source: Front Pharmacol. 2023 Mar 22;14:1107494. doi: 10.3389/fphar.2023.1107494 (PMC10073577; doi:10.3389/fphar.2023.1107494)
Supplement: Supplementary file 1 [file Table1.DOCX]

Supplementary Material

# Supplementary Tables

Supplementary Table 1. lists of the laxatives evaluated in this study.

| Drug name of laxatives |
| --- |
| Magnesium oxide |
| Senna glycosides |
| Daikenchuto |
| Mashiningan |
| Junchoto |
| Jizusoippo |
| Tokakujokito |
| Bofutsushosan |
| Choijokito |
| Daiokanzoto |
| Jidabokuippo |
| Tsudosan |
| San'oshashinto |
| Daijokito |
| Inchinkoto |
| Otsujito |
| Daisaikoto |
| Daiobotampito |
| Keishikashakuyakudaioto |
| Crude daio |

Supplementary Table 2. Kampo-containing rhubarb rhizome.

| Medicine | Composition |
| --- | --- |
| Mashiningan | Hemp Fruit, **Rhubarb**, Immature Orange, Apricot Kernel, Magnolia Bark, Peony Root |
| Junchoto | Hemp Fruit, **Rhubarb**, Immature Orange, Apricot Kernel, Magnolia Bark, Peony Root |
| Jizusoippo | Cnidium Rhizome, Atractylodes Lancea Rhizome, Forsythia Fruit, Lonicera Leaf and Stem, Saposhnikovia Root, Glycyrrhiza, Schizonepeta Spike, Safflower, **Rhubarb** |
| Tokakujokito | Peach Kernel, Cinnamon Bark, **Rhubarb**, Glycyrrhiza, Anhydrous Mirabilitum |
| Bofutsushosan | Aluminum Silicate Hydrate with Silicon Dioxide, Scutellaria Root, Glycyrrhiza, Platycodon Root, Gypsum, Atractylodes Rhizome, **Rhubarb**, Schizonepeta Spike, Gardenia Fruit, Peony Root, Cnidium Rhizome, Japanese Angelica Root, Mentha Herb, Saposhnikovia Root and Rhizome, Ephedra Herb, Forsythia Fruit, Anhydrous Sodium Sulfate, Ginger |
| Choijokito | **Rhubarb**, Glycyrrhiza, Anhydrous Sodium Sulfate |
| Daiokanzoto | **Rhubarb**, Glycyrrhiza |
| Jidabokuippo | Cinnamon Bark, Cnidium Rhizome, Nuphar Rhizome, Quercus Bark, Glycyrrhiza, **Rhubarb**, Clove |
| Tsudosan | Immature Orange, **Rhubarb**, Japanese Angelica Root, Glycyrrhiza, Safflower, Magnolia Bark, Sappan Wood, Citrus Unshiu Peel, Akebia Stem, Anhydrous Sodium Sulfate |
| San'oshashinto | Scutellaria Root, Coptis Rhizome, **Rhubarb** |
| Daijokito | Magnolia Bark, Immature Orange, **Rhubarb**, Anhydrous Sodium Sulfate |
| Inchinkoto | Artemisia Capillaris Flower, Gardenia Fruit, **Rhubarb** |
| Otsujito | Japanese Angelica Root, Bupleurum Root, Scutellaria Root, Glycyrrhiza, Cimicifuga Rhizome, **Rhubarb** |
| Daisaikoto | Bupleurum Root, Pinellia Tuber, Scutellaria Root, Peony Root, Jujube, Immature Orange, Ginger, **Rhubarb** |
| Daiobotampito | Benincasa Seed, Peach Kernel, Moutan Bark, **Rhubarb**, Anhydrous Sodium Sulfate |
| Keishikashakuyakudaioto | Hemp Fruit, **Rhubarb**, Immature Orange, Apricot Kernel, Magnolia Bark, Peony Root |
| Crude daio | **Rhubarb** |

All crude drugs are listed in the Japanese Pharmacopeia 18^th^ Edition.

Supplementary Table 3. ICD-10 codes for MCMs.

| MCMs | ICD-10 code |
| --- | --- |
| Nervous system | Q00-Q07  excluding minor CMs; Q10 |
| Eyes, ears, face, and neck | Q10-Q18  excluding minor CMs; Q162, Q17–Q182, Q184–Q189 |
| Circulatory system | Q20-Q28  excluding minor CMs; Q250, Q270 |
| Respiratory system | Q30-Q34 |
| Cleft lip and cleft palate | Q35-Q37 |
| Digestive system | Q38-Q45  excluding minor CMs; Q381 |
| Genital organs | Q50-Q56  excluding minor CMs; Q515, Q516, Q520–Q527, Q53 |
| Urinary system | Q60-Q64 |
| Musculoskeletal system MCMs and deformations | Q65-Q79  excluding minor CMs; Q664–Q666, Q69 |
| Other | Q85-Q89 |

Abbreviations: ICD-10 = International Classification of Diseases, 10^th^ Revision; MCM = Major congenital malformations.
